# Towards Induction of Angiogenesis in Dental Pulp Stem Cells Using Chitosan-Based Hydrogels Releasing Basic Fibroblast Growth Factor

**DOI:** 10.1155/2022/5401461

**Published:** 2022-02-14

**Authors:** Baharak Divband, Bahareh Pouya, Mehdi Hassanpour, Mahdieh Alipour, Roya Salehi, Reza Rahbarghazi, Sahriar Shahi, Zahra Aghazadeh, Marziyeh Aghazadeh

**Affiliations:** ^1^Dental and Periodontal Research Center, Tabriz University of Medical Sciences, Tabriz, Iran; ^2^Faculty of Dentistry, Tabriz University of Medical Sciences, Tabriz, Iran; ^3^Stem Cell Research Center, Tabriz University of Medical Sciences, Tabriz, Iran; ^4^Department of Biochemistry and Clinical Laboratories, Faculty of Medicine, Tabriz University of Medical Sciences, Tabriz, Iran; ^5^Department of Medical Nanotechnology, Faculty of Advanced Medical Sciences, Tabriz University of Medical Sciences, Tabriz, Iran; ^6^Department of Applied Cell Sciences, Faculty of Advanced Medical Sciences, Tabriz University of Medical Sciences, Tabriz, Iran; ^7^Department of Endodontics, Dental School, Tabriz University of Medical Sciences, Tabriz, Iran; ^8^Department of Oral Medicine, Faculty of Dentistry, Tabriz University of Medical Sciences, Tabriz, Iran

## Abstract

**Introduction:**

Chitosan is a natural biopolymer that attracted enormous attention in biomedical fields. The main components of regenerative endodontic procedures (REPs), as well as tissue engineering, are scaffolds, stem cells, and growth factors. As one of the basic factors in the REPs is maintaining vascularization, this study was aimed at developing basic fibroblast growth factor- (bFGF-) loaded scaffolds and investigating their effects on the angiogenic induction in human dental pulp stem cells (hDPSCs).

**Methods:**

Poly (*ε*-caprolactone) (PCL)/chitosan- (CS-) based highly porous scaffold (PCL/CS) was prepared and evaluated by scanning electron microscopy (SEM) and Fourier transform infrared spectroscopy (FTIR) analyses. The adhesion and survival potency of seeded cells were assessed by SEM and MTT assays, respectively. The amount of angiogenic markers was investigated in gene and protein levels by real-time PCR and western blotting assays, respectively.

**Results:**

Based on our findings, the SEM and FTIR tests confirmed the appropriate structure of synthesized scaffolds. Besides, the adhesion and survival rate of cells and the levels of VEGFR-2, Tie2, and Angiopoietin-1 genes were increased significantly in the PCL/CS/bFGF group. Also, the western blotting results showed the upregulation of these markers at protein levels, which were considerably higher at the PCL/CS/bFGF group (*P* < 0.05).

**Conclusions:**

On a more general note, this study demonstrates that the bFGF-loaded PCL/CS scaffolds have the potential to promote angiogenesis of hDPSCs, which could provide vitality of dentin-pulp complex as the initial required factor for regenerative endodontic procedures.

## 1. Introduction

Chitosan (CS) is a natural mucopolysaccharide with suitable and fundamental properties required for tissue engineering. This biomaterial is extracted from chitin with low immunogenicity, biodegradability, biocompatibility, nontoxicity, osteoinductivity, hemostasis, sustained drug delivery, and bacteriostatic characteristics that make it suitable for medical application [1–5]. In dentistry, this biomaterial has been evaluated in various studies, especially for the bone regeneration, periodontal, and dentin-pulp complex [6–9]. Managing damaged pulp-dentin complex in immature teeth with open apices is considered a challenging clinical situation for endodontic specialists [10, 11]. Apexification is a definitive treatment in these cases that has some drawbacks, including difficulty in handling and placing the barriers, lack of continuity of physiologic root development and apex closure, and increased possibility of future root fractures [12]. Recently, regenerative endodontic procedures (REPs) have been introduced as a novel treatment approach in the dentistry field. The aim of REPs is remodeling of damaged dentin-pulp complex, including connective tissue, vascularization, and innervation of the pulp tissue and surrounding dentin [13–15]. The pulp is a highly vascularized tissue with infiltration of blood vessels and nerve bundles. The regeneration of this tissue is more challenging than other tissues due to some anatomical factors, including minimal collateral blood supply. Moreover, the angiogenesis of the pulp tissue is more complex due to this architecture [16]. However, this physiological procedure is the major part of vascularization that is essential for efficiently transporting various nutrients, chemokines, inflammatory cells, and cytokines. Therefore, the healing ability of injured pulp, as well as other tissues, depends on angiogenesis, which is incorporated with different growth factors [3, 17–19].

Generally, the successful outcome of REPs depends on contributing three main components of tissue engineering, including stem cells, three-dimensional scaffolds, and growth factors [20]. Stem cells have unique properties and have been found as undifferentiated cells exposed to environmental signals. These cells show a high proliferation ability and self-renewal capacity that differentiate into different cell lineages [21]. Recent findings have suggested that permanent tooth pulp (DPSC), periodontal ligament (PDL), deciduous dental pulp, and apical papillae contain a mass of stem cells [22]. For REPs, the dental pulp is considered a rich reservoir of progenitor stem cells. The discovery of DPSC has introduced a new field of research in dentistry, providing opportunities for the regeneration and replacement of oral and dental tissues [23].

Scaffolds are the other essential components of REPs to overcome the limitations of common traditional treatments used in dentistry [24, 25]. Three-dimensional scaffolds with interconnected porous structures provide an appropriate microenvironment for local distribution of cells, transportation of nutrients and wastes, and infiltration of new vessels. The scaffold should be biocompatible and could resemble the functions of the natural extracellular matrix (ECM). Scaffolds in the regenerative endodontic could be fabricated from natural polymers, synthetic polymers, and a combination of these polymers [26]. CS has high biocompatibility properties and induces the mineralization process; however, as well as other natural polymers, it has relatively low mechanical strength [14, 27, 28]. In order to improve the mechanical characteristics, this natural polymer could be blended with appropriate synthetic polymers such as poly (*ε*-caprolactone) (PCL) [9].

The formation and infiltration of new vessels depend on getting signals from angiogenic growth factors, particularly vascular endothelial growth factor (VEGF). This growth factor elevates endothelial cell differentiation and proliferation potency and improves tube formation, mobilization, and recruitment of endothelial progenitor cells [3]. The basic fibroblast growth factor (bFGF) is another essential growth factor that regulates the angiogenesis process. It also increases the proliferation of progenitor mesenchymal cells in a time- and dose-dependent manner [29]. It is indicated that bFGF regulates the expression of genes and proteins involved in the angiogenesis procedure with stimulating proliferation and differentiation in DPSCs, which has a vital role in the healing and regeneration of injured dentin-pulp complex [30]. Angiogenesis is a basis for engineering complex tissues of mesenchymal stem cells. Therefore, it is essential to develop various strategies for accelerating the angiogenesis potential and tissue perfusion [30, 31]. Despite the numerous studies on the vascularization of the dentin-pulp complex, there is no applicable and efficient REP-based product available for dentists. Designing and fabricating cost-benefit, easy handling, biocompatible, and biodegradable scaffolds releasing growth factors are considered attractive appliances for REPs [32]. Although the bFGF growth factor could potentially induce an angiogenesis, it has been shown that the direct injection of bFGF protein might not lead to remarkable therapeutic effects in host tissues [33]. Therefore, it seems that incorporating an appropriate FDA-approved carrier with this growth factor could be a great idea for potential future applications. Thus, in the current study, as an initial step, we aimed to evaluate the expression of angiogenic genes and proteins in hDPSCs seeded on a PCL/CS polymeric scaffold administered with the bFGF growth factor.

## 2. Methods

### 2.1. Materials

*ε*-Caprolactone, glutaraldehyde (25%), isopropanol, SnO_2_, and CS (degree of deacetylation 85% and Mw = 100, 000–300,000) were obtained from Sigma-Aldrich Co. (Steinem, Germany). Dimethyl sulfoxide (DMSO), dichloromethane, chloroform, acetic acid, and formic acid were purchased from Merck Chemical Co. (Darmstadt, Germany). Also, the bFGF growth factor was obtained from Merck Chemical Co. (Darmstadt, Germany, Cat no. 11123149001).

Phosphate-buffered saline (PBS), trypsin, fetal bovine serum (FBS), high glucose Dulbecco's modified Eagle's medium (DMEM/HG), and penicillin/streptomycin were obtained from Gibco BRL Life Technologies. 3-(4,5-Dimethylthiazol-2-yl-2, 5-diphenyltetrazolium bromide) (MTT) (Carlsbad, CA, USA) and TRIzol buffer (Cat No. 15596-026) were obtained from Invitrogen. DNase1 kit (Cat No. en0521) was provided from FermenTaz (Thermo Fisher Scientific, USA). cDNA synthesis kit and SYBR Green PCR Master Mix were provided by Yekta Tajhiz Azma Company (Cat No. YT4500, Tehran, Iran).

### 2.2. Preparations of Scaffolds

PCL polymers were fabricated using ring-opening polymerization of *ε*-caprolactone (*ε*-CL) in the presence of tin(II) 2-ethylhexanoate as a catalyst at 130°C according to the protocol published previously [34]. For this purpose, 10.0 g of *ε*-CL and 0.1 g (1 wt%) of Sn(Oct)_2_ were added to a 50 ml round-bottom flask. The suspension was heated to 130°C while being stirred in a nitrogen atmosphere for 6 h. The obtained polymer was dissolved in dichloromethane and precipitated in excess of cold diethyl ether to remove the unreacted monomers and excess catalyst. Dialysis was performed for further purification in a 2000-mesh dialysis bag for 3 days until all impurities, remaining probable monomer, and the catalyst residue were removed. The polymer was later dried in a freeze dryer. The PCL polymer was obtained with a molecular weight of 4.5 kDa (obtained by gel permeation chromatography (GPC Agilent 110) using THF as solvent). Then, the PCL/CS scaffold was prepared as follows with a weight ratio of 70 to 30 (PCL/CS). The PCL polymer and CS were dissolved in formic acid and acetic acid, respectively. These two solutions were later mixed in the homogenizer at 20 × 10^3^ rpm. Furthermore, polyvinyl alcohol 1% was added during the homogenization process to prevent the two phases of the mixture as a surfactant. The resulting mixture was then heated at 10°C above the polymer's melting point for 10 min. The solution was then quickly transferred into an ice water solution (8°C) and remained there for 5 min. The sample was then transferred to -80°C and then to a dryer 48 h later.

### 2.3. Fourier Transform Infrared Spectroscopy (FTIR) Assay

The FTIR was used to examine intermolecular interaction between the components of the scaffolds. For FTIR evaluation, one sample of fabricated scaffolds was first poured into a uniform powder with a ratio of one to one hundred pure KBr, and then, the powder was placed in special tubes. After that, the test was performed with the resolution of 4 cm^−1^ at the spectral range of 400 and 4000 cm^−1^ by Tensor 27 (Bruker, Germany).

### 2.4. Isolation, Characterization, and Culture of hDPSCs

The procedures of isolation, characterization, and culture of hDPSCs were performed according to our recent publication [35]. Briefly, hDPSCs were isolated from permanent third molars extracted due to the orthodontic treatment plan. The donor was a 24-year-old female who signed the written consent form after being informed about the study's objective. At the third passage, these cells were determined and characterized by fluorescence-activated cell sorting; cultured in the culture medium containing DMEM/HG, 10% FBS, and 1% penicillin/streptomycin as an antibiotic agent; and incubated at 37°C in an 85-95% humid atmosphere containing 5% CO_2_. After reaching to 80% confluency, the PCL/CS/bFGF scaffolds were seeded in 96-well and 6-well plates. The exhausted culture medium was replaced every three days. All experimental protocols were approved by the Stem Cell Research Center, Tabriz University of Medical Sciences (ethical code: IR.TBZMED.REC.1396.195).

### 2.5. SEM Imaging

The surface morphology and structure of the PCL/CS scaffolds with and without hDPSCs were evaluated by SEM 7 days after seeding. Before assessing, hDPSCs were fixed in 2.5% glutaraldehyde on the scaffolds as described recently [34]. After fixation, the hydrogels containing hDPSCs were dehydrated using a graded series of alcohol concentrations (50, 70, 90, and 100%) [36]. Afterward, scaffolds with and without stem cells were cut into 3 specimens and coated by a thick gold layer. FE-SEM 1430 VP (MIRA3 FEG-SEM-TESCAN, Czech) was applied to characterize these samples.

### 2.6. Loading of bFGF

Loading of bFGF on fabricated scaffolds was done before molecular tests by swelling 0.06 g of each fabricated hydrogel in the PBS buffer solution with 0.3 *μ*g/ml of bFGF at 4°C for 3 days. The volume used was adjusted to load 0.1 *μ*g bFGF per mg of the synthesized hydrogel [37, 38].

### 2.7. Cell Survival Assay

We performed MTT assay to evaluate the survival rate of hDPSCs by estimation of metabolic activity of these cells as described in standard protocols [39]. For this purpose, three 96-well plates were prepared to evaluate the metabolic activity of cells with scaffold as a case group and without facing hydrogels as a control group. Firstly, 50 *μ*l of hydrogels was added to the case wells. Each plate was exposed to UV light for disinfection for 20 minutes. Then, 5 × 10^3^ cells were cultured on each PCL/CS/bFGF scaffold in the 96-well plates. Also, the exact numbers of cells were cultured in wells without scaffolds considered as a control group. The metabolic activity of cells was evaluated 24, 48, and 72 h after the seeding of hDPSCs on the PCL/CS/bFGF scaffold (six specimens for each day). Briefly, 5 mg/ml MTT solution was added to each well, and then, the plates were incubated for 4 h in physiological conditions and darkness in the incubator. After that, DMSO replaced the MTT, and dissolved formazan crystals were measured by an ELISA reader at 570 nm, and the percent of metabolically active cells was estimated according to the intensity of colorimetric changes.

### 2.8. DAPI Staining

For more effective evaluation, the DAPI staining test was performed. This test could help detect both the vital and the attached cells to the hydrogel. At the fourth passage, hDPSCs were seeded on hydrogels at a density of 5 × 10^3^ in two groups containing PCL/CS and PCL/CS/bFGF. In the mentioned days as MTT assay (1, 2, and 3), the samples were stained with DAPI (4′,6-diamidino-2-phenylindole) to evaluate the scaffold's effects on hDPSC survival rate. The samples were washed with PBS three times and then were fixed using 4% paraformaldehyde for 10 minutes. Then, the samples were rewashed with PBS and treated with Triton X-100 to improve cell permeability. Finally, the samples were washed with PBS three more times and were stained with DAPI (3000 nm) (5 minutes). The samples were imaged via Cytation™ system (BioTek, Winooski, USA), and ImageJ software was used for detection of fluorescence intensity.

### 2.9. Angiogenic Gene Expression Analysis by qRT-PCR

The number of 1 × 10^6^ of hDPSCs was seeded on fabricated scaffolds located on 6-well plates (one scaffold on each plate). For the control group, 1 × 10^6^ cells were seeded in 6-well plates without scaffolds. After 7 days, the total RNA was extracted from each template (containing scaffolds and cells) and a control subject (scaffold-free) using Ambion TRIzol buffer as described before (40). After omitting any DNA contamination using the DNase1 kit, 1 *μ*g of total RNA was used for cDNA synthesis according to the manufacture's instruction. For quantitative real-time PCR (qRT-PCR) reaction, synthesized cDNA (1 *μ*l), SYBR Green Master Mix (7 *μ*l), designed primers (1 *μ*l), and DEPC water (5 *μ*l) were mixed as described previously [40]. The qRT-PCR was performed by a Roche LightCycler® 96 Instrument, and fold change values were calculated using the 2^(-*ΔΔ*Ct)^ method with normalization to *β*-actin housekeeping gene. The list of primers, including VEGFR-2, Tie-2, Angiopoietin-1, and *β*-actin, is outlined in Table 1.

### 2.10. Angiogenic Protein Expression Analysis by Western Blotting

Western blot analysis was conducted to evaluate the expression of angiogenic proteins in hDPSC cells seeded on the PCL/CS/bFGF scaffolds. For this test, 1 × 10^6^ of hDPSCs were seeded on the PCL/CS/bFGF scaffolds which were located on 6-well plates. Also, for the control group, 1 × 10^6^ cells were seeded on 6-well plates without scaffolds. After 7 days, cells were lysed in ice-cold cell lysis buffer solution (NaCl, NP-40, and Tris–HCl) containing cocktail enzyme inhibitors. Then, the solutions were sonicated and centrifuged at 14,000 × *g* for 20 min. The total protein contents were measured in the supernatant by Picodrop spectrophotometer system (Model No. PICOPET01, Serial No. 000212/1) and resolved by the SDS-PAGE method as described previously [40]. The samples were incubated overnight at 4°C in the following primary antibody solution: VEGFR-2 (Cat No. ab39256, Abcam Company), Angiopoietin-1 (Cat No. sc-517593, Santa Cruz Biotechnology, Inc.), Tie-2 (Cat No. sc-293414, Santa Cruz Biotechnology, Inc.), and *β*-actin (Cat No. sc-47778, Santa Cruz Biotechnology, Inc.). After that, the samples were incubated with secondary HRP-conjugated anti-IgG antibody (Cat No. sc-2357, Santa Cruz Biotechnology, Inc.) for 1 h at room temperature. According to the manufacturer's instructions, the immunoreactive blots were detected by the ECL plus solution kit (Bio-Rad) to visualize the reactive proteins on the blots.

### 2.11. Statistical Analysis

Statistical analyses were performed using Prism software (version 8.0, GraphPad, San Diego, CA, USA). The normality and homogeneity of the data distribution were analyzed by the Kolmogorov-Smirnov test. The continuous values with normal distribution were reported as mean ± SD and analyzed by Student's *t*-test using Prism software (version 8.0, GraphPad, San Diego, CA, USA). *P* value ≤ 0.05 was considered statistically significant. All experiments were carried out in triplicate.

## 3. Results

### 3.1. Structural Analysis

**Figure 1:**
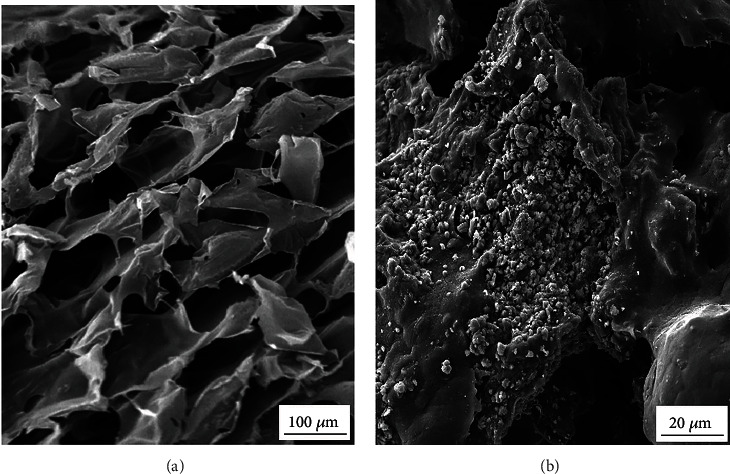
Surface morphology of (a) PCL/CS scaffolds and (b) adhesion and spreading of hDPSCs seeded on PCL/CS scaffolds with various magnifications.

The SEM image of the PCL/CS scaffolds showed macroporous structures with uniform shape and structure (Figure 1(a)). Moreover, as is shown in Figure 1(b), the human dental pulp stem cells were adhered and spread to the scaffold structure.

**Figure 2:**
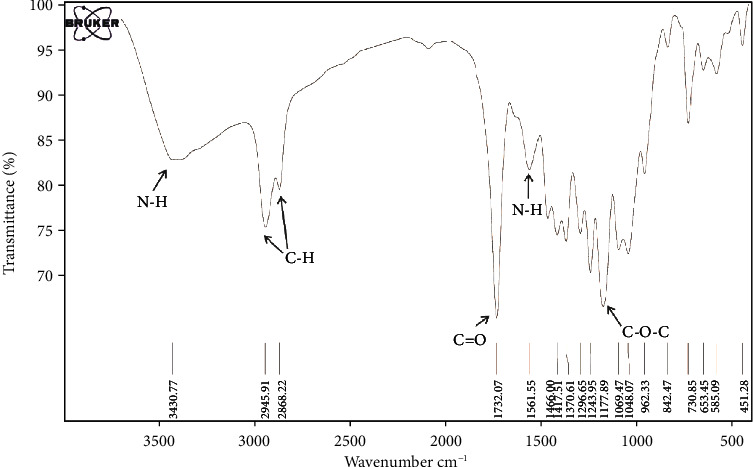
FTIR spectra of PCL/CS scaffolds.

The FTIR spectra of the PCL/CS scaffolds are demonstrated in Figure 2. Peaks at 3430 cm^−1^ were related to NH stretching of the primary amino groups of CS, and OH stretching vibration was related to both PCL and CS parts of the scaffold. Moreover, 1561 cm^−1^ (NH bending of amides I and II) of CS, 1466 cm^−1^ (CH), and 1096 cm^−1^ (COC stretching vibration) existed in the PCL/CS curve. The stretching vibration band of C=O at 1732 cm^−1^ was assigned as a characteristic peak of ester related to the PCL segment of the scaffold. Moreover, the stretching vibration bands of CH_2_ and C-O-C are located at 2868-2945 cm^−1^ and 1177-1243 cm^−1^, respectively. The presence of all characteristic peaks of CS and PCL in the FTIR spectra proved the blend scaffold's successful preparation.

### 3.2. MTT Assay and DAPI Staining

**Figure 3:**
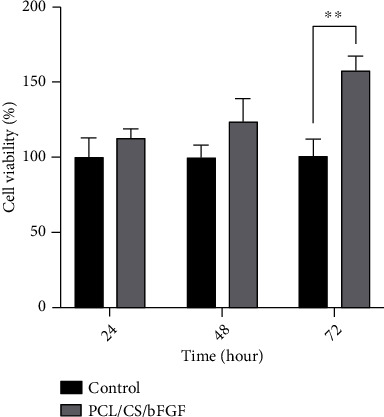
The viability and survival rate of hDPSCs seeded on PCL/CS/bFGF scaffold. ^∗∗^*P* ≤ 0.01.

The effect of the PCL/CS/bFGF scaffold on the metabolic activity and viability of DPSCs was evaluated by MTT assay. The results in Figure 3 demonstrated an increased in viability of cells in the case groups. To evaluate the survival rate of cells in each group, the digit related to the control group was considered as 100%, and the percentage of viability in case groups was evaluated by division of case group digit to the control group. Compared to the control group, the percentage of viable cells (metabolically active cells) was 113%, 124%, and 158% after 24, 48, and 72 hours, respectively. However, the increases in the percentage of viable cells were not significant after 24 and 48 hours; the results confirmed a lack of cytotoxic effect of hydrogels. This increase was significant at 72 hours; the cell survival rate on the PCL/CS/bFGF scaffolds was significantly higher than in the control group. These results showed that the fabricated PCL/CS/bFGF scaffolds in the current study were not cytotoxic. Moreover, the hydrogel improved the metabolic activity of cells over time. Our data was in line with similar studies that evaluated the proliferative effect of VEGF-releasing hydrogels on DPSCs [41, 42].

**Figure 4:**
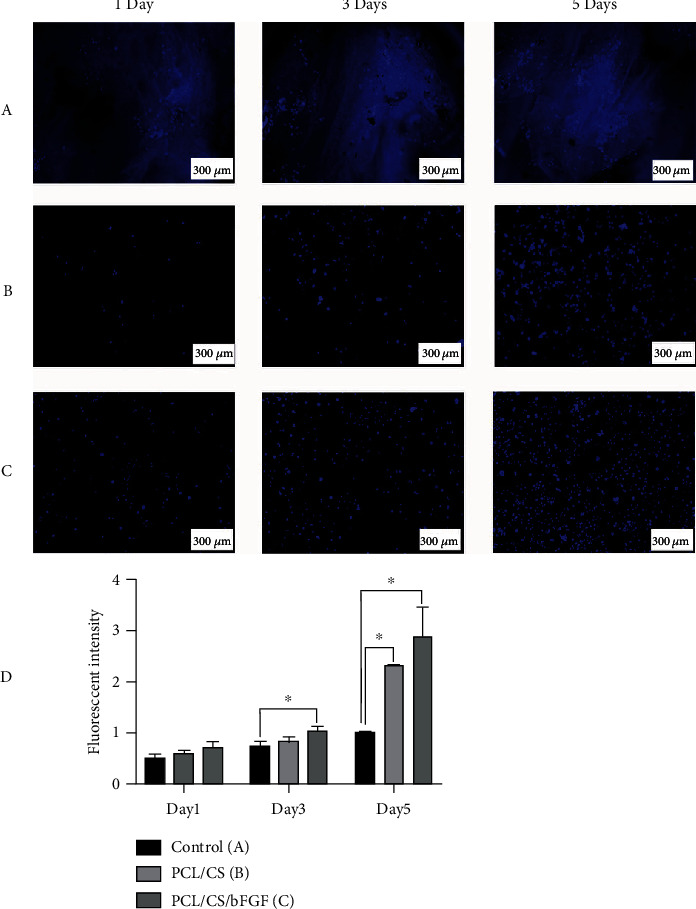
DAPI-stained DPSCs seeded on hydrogels at different times. (a) Images taken with a limited source of light showed the hydrogel in the background. Stained nuclei on the PCL/CS hydrogels (b) and PCL/CS/bFGF hydrogels (c) were increased in number during the time. (d) The fluorescent intensity in the same groups at days 1, 3, and 5. The intensity is significantly increased in wells containing hydrogel at day 5 (^∗^*P* ≤ 0.0).

According to the DAPI-stained images (Figure 4), the number of detectable fluorescent nuclei increased with time passing, and this rise was significant in the groups containing hydrogel at day 5. Despite the softness of the hydrogels, which made the process of staining difficult, there was evidence of the possibility of cell penetration and proliferation into the scaffold. As a result, the attached cells inside the hydrogels were not removed during the multiple washing processes (Figure 4(a)). In the first line of Figure 4, the microscope's light is not filtered completely, so the hydrogel is visible in the background. The nuclei of DPSCs located inside the hydrogels are detectable. In the second and third lines of this figure (Figures 4(b) and 4(c)), the darkness increased, which helps detect the nuclei of vital cells. To quantify DAPI-positive cells in each group, ImageJ software was used. The fluorescence intensity of the blue color which was a reflection of stained vital nuclei was detected and compared in mentioned times (Figure 4(d)). According to the results, this intensity increased by time and was significant in the groups containing hydrogel on the last day of assessment. Furthermore, the number of stained nuclei was significantly raised in the presence of PCL/CS/bFGF hydrogel both in the third and fifth day. However, there was no significant difference in the groups of DPSCs exposed to scaffolds releasing bFGF or base of the hydrogels.

### 3.3. Relative Gene Expression of Angiogenic Markers

**Figure 5:**
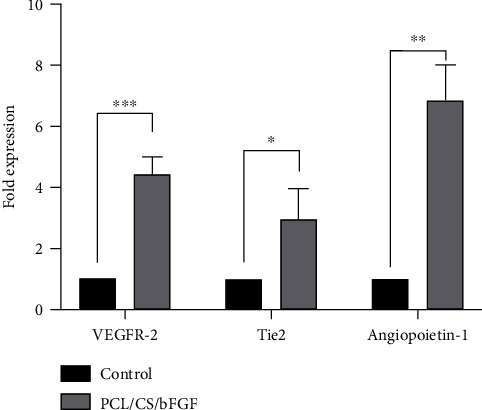
Expression levels of VEGFR-2, Tie-2, and Angiopoietin-1 genes in hDPSCs seeded on PCL/CS/bFGF scaffolds after 7 days. The seeding of hDPSCs on these scaffolds significantly increased angiogenic gene expression. ^∗^*P* ≤ 0.05, ^∗∗^*P* ≤ 0.01, and ^∗∗∗^*P* ≤ 0.001.

The expression levels of angiogenic genes in hDPSCs seeded on PCL/CS/bFGF scaffolds were evaluated on day 7. As shown in Figure 5, the expression of all evaluated angiogenic genes was significantly upregulated in the presence of the PCL/CS/bFGF scaffold. The expression of VEGFR-2, Tie-2, and Angiopoietin-1 showed 4.4-fold, 2.9-fold, and 6.82-fold increases compared to the control group, respectively. The level of expression of Angiopoietin-1 was elevated more than other studied genes in the presence of the PCL/CS/bFGF scaffolds.

### 3.4. Expression of Angiogenic-Related Proteins

**Figure 6:**
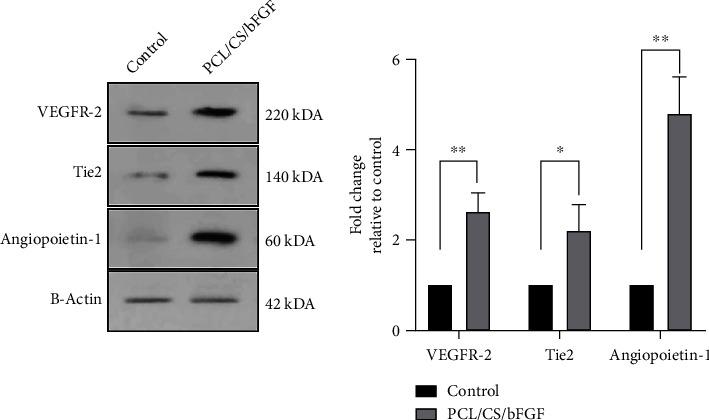
Expression levels of VEGFR-2, Tie-2, and Angiopoietin-1 in hDPSCs seeded on PCL/CS/bFGF scaffolds by western blotting after seven days. The seeding of hDPSCs on these scaffolds significantly increased the expression of evaluated angiogenic markers in protein levels. ^∗^*P* ≤ 0.05 and ^∗∗^*P* ≤ 0.01.

In order to evaluate the angiogenic induction ability of the PCL/CS/bFGF scaffolds in protein levels, the expression levels of VEGFR-2, Tie-2, and Ang-1 in seeded hDPSCs were measured. As shown in Figure 6, all 3 angiogenic-related proteins were significantly upregulated in the PCL/CS/bFGF group compared to the control group after 7 days. The expression levels of Ang-1 were the highest among the 3 markers.

## 4. Discussion

In the current study, the effect of chitosan-based scaffolds containing bFGF growth factor was investigated on the extent of angiogenesis of hDPSCs seeded on the PCL/CS/bFGF scaffold in *in vitro* milieu. bFGF was selected because of the induction of cytokine synthesis in hDPSCs and stimulation of the angiogenic response in the pulp tissue [43]. We isolated these cells from the third molars of a single donor. As we used healthy teeth extracted due to orthodontic treatment, we got a considerable amount of highly proliferative pulp cells and used them as the main supply for this study. However, using more than one donor is usually applied in similar studies [44].

DPSCs can differentiate into angiogenic cells [45]. In the present study, we have been trying to increase this capacity by bFGF-releasing hydrogels to improve the clinical performance of these cells in practice in the future. For this purpose, we focused on the conditions of clinical application of this biomaterial. We defined the groups according to the practical use during dental treatments. Therefore, two groups were developed, the cells exposed with the growth factor as case group and DPSCs without using the hydrogel as the control group, which reflects the routine manner of cells in dental treatments. However, for an exact evaluation, it is suggested to consider the group of basic hydrogel without growth factor in further studies. Previously, scientific manuscripts have reported that hDPSCs can differentiate into endothelial cells by secretion of proangiogenic factors [43, 46, 47]. Effective vascularization is mandatory for the healing of injured pulp tissues. It is well documented that loading angiogenic growth factors on suitable biocompatible biomaterials could enhance pulp tissue healing [3]. In addition to their differentiation potential, these cells have a high proliferation capacity, which makes them attractive to researchers [48]. The amount of available mesenchymal stem cells is an important factor for the regeneration of the dentin-pulp complex [49]. It is a challenging issue for the therapeutic teams to provide available and easy access sources of stem cells for tissue regeneration. Although sometimes this supply is provided by transplantation, it is more conservative and cost-effective to enrich the available cells of the defected region. The pulp tissue contains limited mesenchymal stem cells, which are sensitive to thermal and chemical irritants. The number of these cells is decreased by caries and pulpitis [50]. Therefore, it is a critical point to save and increase the amount of these cells for pulp and dentin regeneration. According to the results of proliferation tests in the current study, there were no cytotoxic effects of the PCL/CS/bFGF scaffold. Moreover, this hydrogel improved the metabolically active cells and percentage of viable DPSCs over time. This elevation in the metabolic activity of cells was significant on the third day. In a similar study, CS hydrogel's effect with sustained VEGF delivery on DPSC proliferation was assessed. According to the results, the proliferation of these cells was increased from day 1 to day 7. This elevation was significant on day 5 and day 7 [41]. In another study, the effect of hydroxyapatite/calcium sulfate scaffold releasing VEGF was assessed on the proliferation of mesenchymal stem cells and human umbilical vein endothelial cells. The authors reported the positive effects of this scaffold on cell proliferation [51]. These findings clarify the critical role of the availability of mesenchymal stem cells for the tissue regeneration process. Our study demonstrated a safe and suitable scaffold, which provides a 3D environment for cell attachment and proliferation. The DAPI staining images revealed the DPSC attachment to the hydrogels, and the MTT assay showed the increase of living cell percentage. This increase in the number of cells was shown in DAPI staining in the presence of hydrogel compared to cells alone. According to the results of ImageJ, the intensity of the fluorescence was elevated in the presence of bFGF.

Chitosan, as a natural polysaccharide, was evaluated for regenerative purposes in dentistry [14]. This polymer demonstrates significant healing properties and modulates the inflammation procedure regulating the function of fibroblasts, inflammatory cells, and macrophages [52, 53]. Functional amino groups in the CS polymer chain make it suitable for drug release [54]. Therefore, different studies loaded various growth factors on chitosan-based scaffolds, including bone morphogenetic proteins (BMP family, such as BMP-2, BMP-6, and BMP-7), VEGFs, transforming growth factor-beta 1 (TGF-*β*1), and bFGF [3, 55–57].

PCL-based synthetic polymers improved the mechanical characteristics of natural polymers such as CS and showed successful results in calcified tissue engineering [58]. In the current study, we used both of these polymers to fabricate porous scaffolds providing a 3D microenvironment for hDPSC adhesion, proliferation, and differentiation. The SEM figures proved the appropriate structure of fabricated PCL/CS scaffold for adhesion and proliferation of human dental pulp stem cells. The FTIR test is used to confirm the chemical composition of polymers in the scaffold structure [59]. The combination of PCL and CS polymers in fabricated scaffold was proved by this test in the current study.

The other important part of angiogenic induction is an appropriate growth factor, which can directly induce the endothelial phenotypes on hDPSCs. Generally, in the procedure of tissue regeneration, growth factors are considered as essential signaling molecules to initiate the cells for provoking a determined cellular response in the host tissue and proceeding the specific lineage [60, 61]. Different growth factors are used for angiogenesis in host tissue, including platelet-derived growth factor (PDGF), FGF, TGF, and VEGF [62]. Montesano et al. evaluated the angiogenic effects of bFGF; they confirmed the formation of a vascular network after a week [47]. Seghezzi et al.'s study indicated that FGF-2 leads to the expression of VEGF in vascular endothelial cells through autocrine and paracrine mechanisms [63]. Moreover, this growth factor plays a vital role in provoking and improving damaged tissue repair [64]. The expression of VEGFR-2 as markers of endothelial cells indicates the angiogenic induction of hDPSCs [65]. Combining the VEGFR-2 receptor with its marker significantly increases the differentiation of BMMSCs, ASCs, hDPSCs, and SHED endothelial cells [66]. In the current study, synthesized chitosan-based scaffolds were incorporated with bFGF to induce angiogenesis in hDPSCs. Chitosan-based scaffolds were fabricated and then were immersed in bFGF solution to complete the incorporation of bFGF in the scaffold's structure. Also, the morphology of the prepared scaffolds was evaluated through SEM. The fabricated scaffolds showed a porous structure which plays a significant role in tissue engineering with the rehabilitation of ECM and blood vessel infiltration that provide transportation and exchange of gaseous and nutrients [3]. However, for assessing the exact effect of our hydrogel in animal studies, a study group including the base of hydrogel without a stimulator is preferred.

In a study, Kottakis et al. found that bFGF as a growth factor modulates cell proliferation, migration, and angiogenic differentiation through the NDY1/KDM2B-mir-EZH2 pathway [67]. Moreover, Kikuchi et al. evaluated the effect of the gradual release of FGF-2 on dentin formation in the exposed portion of the pulp [31]. The results revealed that the controlled release of FGF2 led to the establishment of dentin-like structures. In the other study, the addition of FGF-2 and FGF-2/TGF*β*1 to the culture media of DPSCs was evaluated, and the results showed that these factors would induce proliferation and differentiation of hDPSCs [68]. In another study, Shimabukuro et al. evaluated the regenerative effects of FGF-2 on the pulp tissue. The results of this experiment suggested that this factor could increase the proliferation and migration of hDPSCs; therefore, the bFGF-loaded scaffold is indicated as an essential candidate for pulp capping [69]. In 2015, Sagomonyants and colleagues demonstrated that FGF2 as one of the FGF growth factors did not show significant influences on the extent of mineralization but stimulated dramatic enhancement in the expression of DMP1 and DSPP and the number of DMP1-GFP+ and DSPP-Cerulean+ odontoblasts [70]. As mentioned before, the essential part of successful pulp regeneration is revascularization. For this purpose, the presence of hDPSCs and appropriate growth factors is crucial, as well as proper scaffolds [20].

It has been shown that treating the human root canal with collagen scaffold and bFGF improves vascular regeneration and dentin formation compared with usual root canal therapy [71]. Moreover, the effects of this growth factor in migration, proliferation, and differentiation of other tooth-derived stem cells, such as stem cells from the apical papilla (SCAP) or stem cells from human exfoliated deciduous teeth (SHED), demonstrated the same enhancement as well as occurred in hDPSCs [72, 73]. In our study, PCL/CS/bFGF scaffolds increased angiogenic markers of hDPSCs. It is worth noting that VEGFR-2 is a well-known angiogenic marker that mediates most of the downstream properties of VEGF in angiogenesis [74, 75].VEGF induced VEGFR2 internalization, which plays a crucial role in activation, and downstream signaling, which is essential for stimulating angiogenesis [76]. Our results indicated the upregulation of this marker in hDPSCs both in gene and protein levels. In another study, Kim et al. isolated stem cells from the inflamed pulp of the deciduous tooth and assessed the effect of bFGF on the regeneration ability of inflamed SHED. They observed that the inflamed pulp cells treated with bFGF had enhanced proliferation and migration ability. However, it has a diminishing effect on cell differentiation, though differentiation and dentin formation increased in the ectopic placement of these cells [77]. All of the mentioned studies confirmed the positive effect of bFGF in the angiogenic process alone. However, in the present study, the administration of this growth factor with chitosan-based scaffold as a carrier for bFGF revealed promising results regarding the angiogenic induction in hDPSCs. According to our results, the expression of angiogenic markers (Angiopoietin-1, Tie2, and VEGFR2) in DPSCs was elevated by using PCL/CS/bFGF hydrogels. The elevation of Angiopoietin-1 was even more than other studied genes. As Angiopoietin-1 is an essential protein for endothelial cell survival, vascular branching, and pericyte recruitment [78], this scaffold seems to promote pulp and tooth regeneration. In the present experiment, the first step was taken to determine the effectiveness of bFGF-loaded scaffolds on angiogenic induction, which is essential for the regeneration of the dentin-pulp complex. However, further *in vivo* investigation is required to approve the clinical application of bFGF-releasing scaffolds.

## 5. Conclusion

Based on the current study results, it could be concluded that the bFGF-loaded PCL/CS scaffolds can promote angiogenesis of hDPSCs, which could provide pulp vitality as the initial required factor of pulp regeneration.

## Figures and Tables

**Table 1:** The sequence of designed primers.

Genes	Sense	Antisense	Tm
VEGFR-2	CCAGCAAAAGCAGGGAGTCTGT	TGTCTGTGTCATCGGAGTGATATCC	60
Tie-2	ATAGGGTCAAGCAACCCAGC	GCTGGTTCTTCCCTCACGTT	60
Ang-1	GGACAGCAGGAAAACAGAGC	CACAAGCATCAAACCACCAT	63
*β*-Actin	AGTGTGACGTTGACATCCGT	TGCTAGGAGCCAGAGCAGTA	60

## Data Availability

The datasets used and/or analyzed during the current study are available from the corresponding author on reasonable request.
